# Lipid transfer proteins and PI4KIIα generate a phosphoinositide-linked proteome

**DOI:** 10.1016/j.jbc.2026.113089

**Published:** 2026-05-05

**Authors:** Noah D. Carrillo, Mo Chen, Poorwa Awasthi, Tianmu Wen, Xiangqin Chen, Giang Vu, Dhruv Brahmbhatt, Michael Herlihy, Benjamin B. Minkoff, Michael Sussman, Vincent L. Cryns, Richard A. Anderson

**Affiliations:** 1 University of Wisconsin Carbone Cancer Center, University of Wisconsin-Madison, School of Medicine and Public Health, Madison, Wisconsin, USA; 2 Department of Medicine, University of Wisconsin-Madison, School of Medicine and Public Health, Madison, Wisconsin, USA; 3 Center for Genomic Science Innovation, University of Wisconsin-Madison, Madison, Wisconsin, USA; 4 Department of Biochemistry, University of Wisconsin-Madison, Madison, Wisconsin, USA

**Keywords:** Akt, cell stress, nucleus, p53, phosphatidylinositol transfer protein, pPhosphoinositide, PI 4-kinase, PI3,4,5P_3_, PI4,5P_2_, PIP_n_-linked proteins

## Abstract

Phosphoinositide (PIP_n_) lipid second messengers in membranes regulate numerous cellular processes. In the cytosol, the phosphatidylinositol (PI) 3-kinase (PI3K)/Akt pathway is scaffolded on IQGAP1 to facilitate the activation of Akt by the synthesis of PI3,4,5P_3_. In the nucleus, PIP_n_ signaling occurs in compartments separate from membranes by stably linking PIP_n_s to nuclear proteins. While several of these proteins have been identified, understanding the extent and impact of protein-linked PIP_n_ signaling warrants further investigation. The tumor suppressor p53, was shown in the companion paper to be regulated by PI transfer proteins (PITPs) and a PI 4-kinase (PI4KIIα), which are required to form p53-PIP_n_ complexes that assemble a nuclear PI3K/Akt pathway. Here we report that class I PITPs (PITPα/β) and PI4KIIα initiate PIP_n_ linkages to many different proteins. PITPα/β and PI4KIIα accumulate in the nucleoplasm in response to stress and are necessary to synthesize nuclear PIP_n_s linked to proteins. These PITPα/β-dependent protein-PIP_n_ complexes are detected by metabolically labeling cells with the PIP_n_ precursor [^3^H]-*myo*-inositol and resist denaturation and SDS-PAGE, indicating that these protein-PIP_n_ complexes represent a putative posttranslational modification. Proteomic and gene set enrichment analysis of proteins that are linked to PI4,5P_2_ reveals an emerging PIP_n_-linked proteome (PIPylome) regulated by PITPα/β and enriched in proteins that play key functional roles in metabolism, cell motility/division, and the DNA damage response. The PIP_n_-linked proteome represents a third messenger signaling paradigm distinct from the canonical membrane-localized pathway whereby linked PIP_n_ messengers regulate protein function.

Phosphoinositide (PIP_n_) lipid second messengers are synthesized in membranes from phosphatidylinositol (PI) by PIP kinases and phosphatases to regulate metabolism, membrane trafficking, motility, cell growth and death (1, 2, 3, 4, 5). Strikingly, the PI 3-kinase (PI3K)/Akt pathway is scaffolded on IQGAP1, which uses PI to synthesize PI3,4,5P_3_ by an ordered assembly of PIP kinases ending with the PI3K-dependent synthesis of PI3,4,5P_3_ that recruits and activates the serine-threonine kinase Akt pathway on membranes (6, 7). PIP_n_ signaling also occurs in non-membranous regions of the nucleus, and these signaling competent PIP_n_s are resistant to detergent extraction, indicating a stable interaction (8, 9, 10, 11, 12), that appears distinct from conventional lipid compartments (4, 13, 14, 15, 16, 17). Consistent with this non-canonical PIP_n_ signaling, six of the seven PIP_n_ isomers (all but PI3,5P_2_), and many PIP kinases and phosphatases, have been reported in non-membranous cellular compartments in the nucleus (4, 9, 18). Several noteworthy PIP_n_-linked proteins have recently been identified, including murine double minute 2 (MDM2) (19), nuclear speckle-targeted PIPKIα-regulated poly (A) polymerase (Star-PAP) (20), Yes-associated protein (YAP) and transcriptional coactivator with PDZ-binding motif (TAZ) (16), nuclear factor erythroid 2-related factor 2 (NRF2) (21), and the tumor suppressor p53 (14). In each case, PIP_n_-linkage is resistant to denaturation/SDS-PAGE and is metabolically labeled with the PI precursor [^3^H]-*myo*-inositol. Furthermore, small heat shock proteins such as αB-crystallin and Hsp27 are recruited to PIP_n_-linked proteins and regulate protein stability and likely other functions. In the nucleus, the regulated assembly/disassembly of PI3,4,5P_3_ linked to p53 by PIP kinases and PTEN, respectively, controls nuclear Akt activation and may be a mechanism for mutant p53’s oncogenic activity (13).

In the companion paper to this manuscript (22), we discovered novel regulators of the p53-PI3K/Akt pathway, namely, the class I (α/β) PI transfer proteins (PITPs) (23, 24) and the PI 4-kinase PI4KIIα (25), which were previously thought to function only in the cytosol. Specifically, we demonstrated that PITPα/β and PI4KIIα interact with p53 in the nucleus and initiate the synthesis of p53-PIP_n_ complexes (22). Given the existence of other PIP_n_-linked proteins, we postulated that PITPα/β and PI4KIIα might be key components of a broader program to link PIP_n_s to other proteins. Here we show that this is indeed the case. PITPα/β and PI4KIIα are required to initiate stable PIP_n_ linkages to a subset of cellular proteins (coined the PIPylome) detected by immunoblotting and metabolic labeling of cells with the PIP_n_ precursor [^3^H]-*myo*-inositol. PI4,5P_2_-linked proteins were affinity purified and identified by mass spectrometry to define an emerging PIPylome of PIP_n_-linked proteins that underscore a novel PIP_n_ signaling paradigm.

## Results

### The nuclear localization of PITPs is regulated by cellular stress

**Figure 1:**
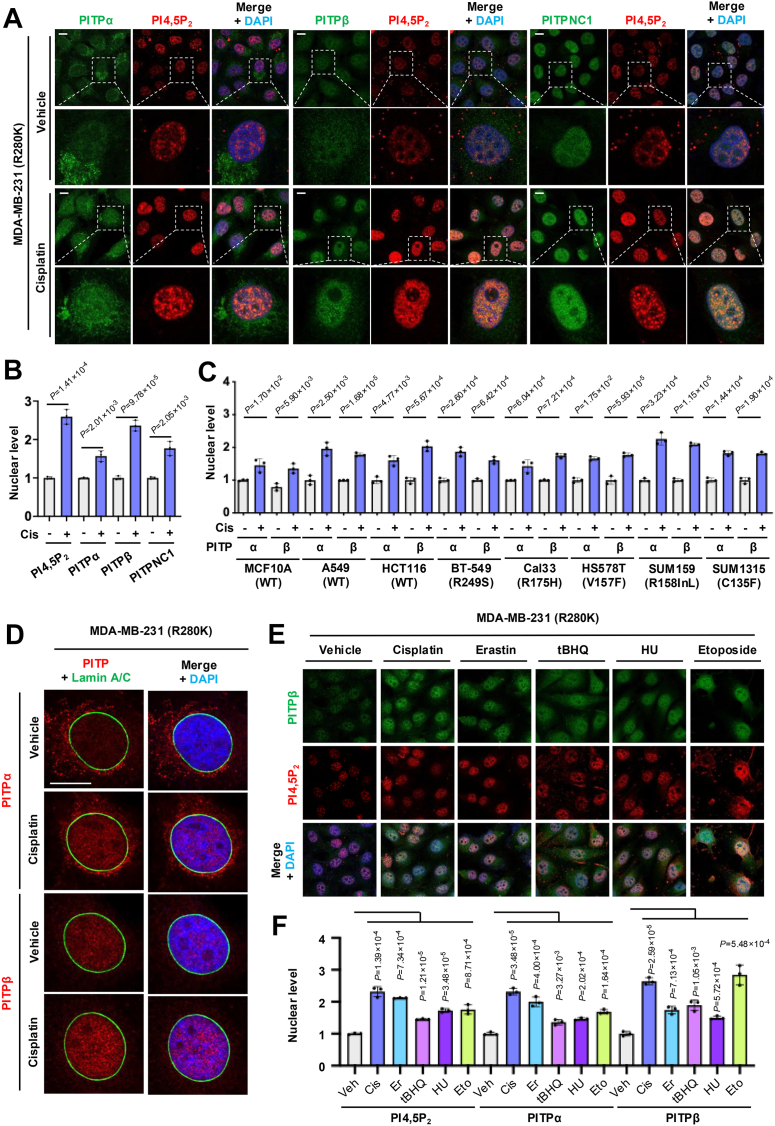
**PITPα and PITPβ accumulate in the nucleus in response to stress.***A* and *B*, confocal images of IF staining against PITPα/PITPβ/PITPNC1 and PI4,5P_2_ in MDA-MB-231 cells treated with vehicle or 30 μM cisplatin for 24 h. The nuclei were counterstained by DAPI. The nuclear levels of PITPs and PI4,5P_2_ were quantified by ImageJ (*B*). n = 3, average values were calculated from 15 cells from each independent experiment. *p* value denotes two-sided paired *t* test. *C*, quantification of IF staining against PITPα/β in wild-type and mutant p53 expressing cells, including MCF10A (WT), A549 (WT), HCT116 (WT), BT-549 (R249S), Cal33 (R175H), HS578 T (V157F), SUM159 (R158InL), and SUM1315 (C135F). Nuclear PITPα and PITPβ levels were quantified by ImageJ. *p* value denotes two-sided paired *t* test. See expanded images in Fig. S1*A*. n = 3, average values were calculated from 10 cells from each independent experiment. *D*, confocal images of IF staining against PITPα or PITPβ overlaid with the nuclear envelope marker Lamin A/C in MDA-MB-231 cells treated with vehicle or 30 μM cisplatin for 24 h. The nuclei were counterstained by DAPI. n = 3 independent experiments. *E* and *F*, MDA-MB-231 cells treated with 30 μM cisplatin, 20 μM erastin, 100 μM tBHQ, 100 μM etoposide, 100 μM hydroxyurea, or vehicle for 24 h. The cells were processed for IF staining against PITPα/β and PI4,5P2. The nuclei were counterstained by DAPI. The nuclear levels of PITPs and PI4,5P2 were quantified by ImageJ (*F*). See expanded images in Fig. S3*E*. n = 3, average values were calculated from 15 cells from each independent experiment. For all graphs, data are presented as the mean ± SD. Scale bar, 5 μm.

The discovery that PITPα/β initiate nuclear p53-PIP_n_ signaling in the companion paper suggests that PITPs may function beyond their established roles in the cytosol (22). To investigate this possibility, we first examined the subcellular localization of the smallest and most homologous members within the PITP family, class I PITPα and PITPβ and class II PITPNC1 (26) by immunofluorescence (IF). Each of these PITPs and PI4,5P_2_ were expressed in the nucleus under basal conditions, and the nuclear levels of each isoform and PI4,5P_2_ were increased by DNA damage (Fig. 1, *A* and *B*). The nuclear translocation of PITPα/β in response to genotoxic stress was observed across all cell lines examined (Figs. 1*C* and S1*A*). Moreover, the nuclear levels of PITPs correlated with nuclear levels of PI4,5P_2_ and over 60% of the IF signal for PITPα/β was in the nucleus after cisplatin treatment (Fig. S1, *B* and *C*). The basal and genotoxic stress-induced nuclear localization of PITPα/β was confirmed by co-IF with lamin A/C to define the nuclear envelope (Figs. 1*D* and S2, *A–D*). This was further validated using 3D sectioning (Fig. S3, *A–D*). Notably, multiple cellular stressors enhanced the nuclear localization of PITPα/β (Figs. 1, *E* and *F* and S3*E*). Taken together, these findings underscore that stress enhances the nuclear localization of PITPα/β.

**Figure 2:**
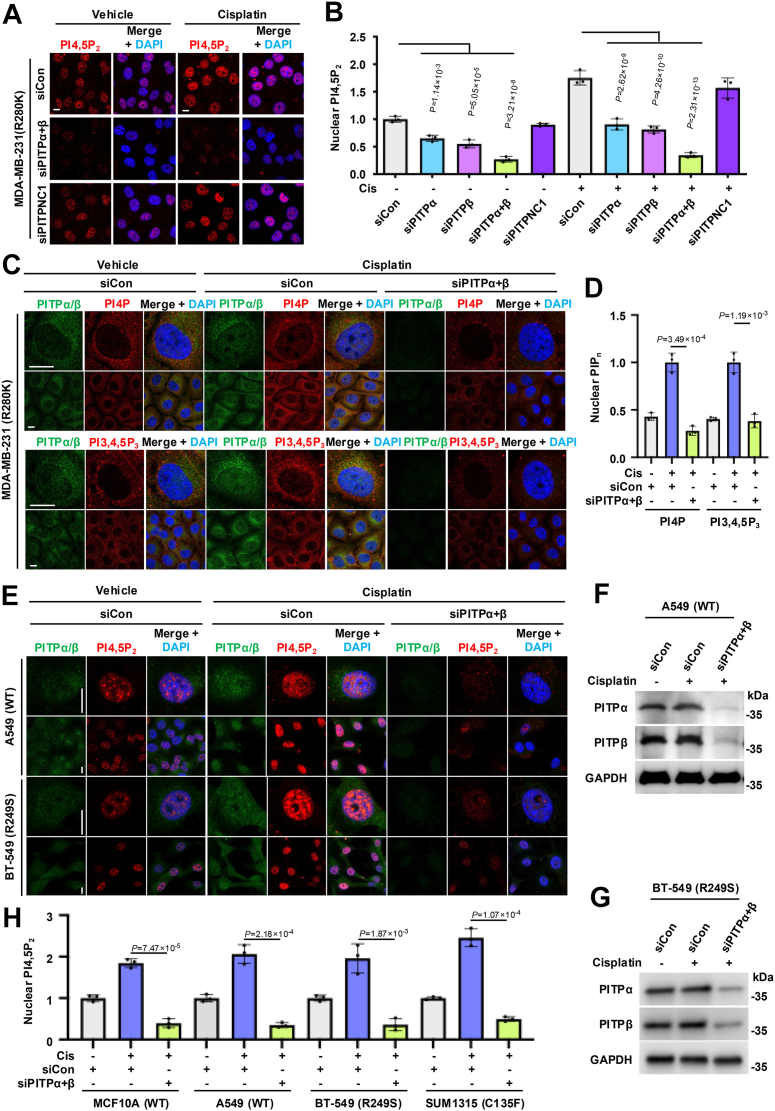
**PITPα/β regulate the nuclear PIP_n_ pool.***A* and *B*, MDA-MB-231 cells were transfected with control siRNAs or siRNAs against PITPα, PITPβ, PITPNC1, or both PITPα and PITPβ. After 24 h, cells were treated with 30 μM cisplatin or vehicle for 24 h before being processed IF staining against PI4,5P_2_. The nuclei were counterstained by DAPI and PI4,5P_2_ levels were quantified by ImageJ (*B*). See expanded images in Fig. S4, *A* and KD validation in Fig. S4. *B* and *C*, n = 3, average values were calculated from 15 cells from each independent experiment. *p* value denotes ANOVA with Bonferroni's multiple comparisons test. *C* and *D*, MDA-MB-231 cells were transfected with control siRNAs or siRNAs against both PITPα and PITPβ. After 24 h, cells were treated with 30 μM cisplatin or vehicle for 24 h before being processed IF staining against PI4P or PI3,4,5P_3_. The nuclei were counterstained by DAPI and PIP_n_ levels were quantified by ImageJ (*D*). n = 3, average values were calculated from 15 cells from each independent experiment. *p* value denotes two-sided paired *t* test. *E–H*, A549 (WT), and BT-549 (R249S) cells were transfected with control siRNAs or siRNAs against both PITPα and PITPβ (*F* and *G*). After 24 h, cells were treated with 30 μM cisplatin or vehicle for 24 h before being processed for IF staining against PI4,5P_2_. The nuclei were counterstained by DAPI, and PI4,5P_2_ levels were quantified by ImageJ (*H*). n = 3, average values were calculated from 15 cells from each independent experiment. *p* value denotes two-sided paired *t* test. For all graphs, data are presented as the mean ± SD. Scale bar, 5 μm.

### PITPs regulate the nuclear PIP_n_ pool

A major enigma in the nuclear phosphoinositide field is the molecular and cellular characterization of PIP_n_ pools in non-membranous compartments (8, 9, 10). PI4,5P_2_ detected by specific monoclonal antibodies form distinctive nuclear foci that colocalize with nuclear speckle markers in regions devoid of membranes and are increased in response to genotoxic stress (4, 8, 13, 14, 27). These findings are reminiscent of the stress-enhanced nuclear localization of PITPα/β that occurs with increased nuclear PI4,5P_2_ (Figs. 1 and S2, *A–D*). Given that PI is the precursor of all PIP_n_s (3), we investigated the role of PITPs in regulating nuclear PIP_n_ pools. Silencing PITPα or PITPβ reduced basal and stress-induced nuclear PI4,5P_2_ levels, while combined knockdown (KD) of both PITPα and PITPβ further decreased nuclear PI4,5P_2_ levels compared to the individual KD (Figs. 2, *A* and *B* and S4*A*). In contrast, PITPNC1 KD had little effect on nuclear PI4,5P_2_ levels (Fig. 2, *A* and *B*). Notably, the combination of PITPβ knock out (KO) in MDA-MB-231^Cas9^ cells and KD of PITPα abrogated PITPα/β IF in these cells, validating antibody specificity (Fig. S4, *B* and *C*). Combined KD of PITPα and PITPβ also suppressed stress-induced nuclear levels of PI4P and PI3,4,5P_3_, although these PIP_n_s are largely cytoplasmic under basal and stressed conditions (Fig. 2, *C* and *D*) as previously reported (28, 29, 30, 31, 32). Combined KD of PITPα and PITPβ robustly inhibited stress-induced nuclear PI4,5P_2_ generation in a panel of human transformed and untransformed cells independent of p53 mutational status (Figs. 2, *E–H* and S4, *D* and *E*). Moreover, the reduction in the stress-induced PI4,5P_2_ levels by combined PITPα and PITPβ KD using distinct siRNAs targeting their 3′-UTR was rescued by expression of PITPα^wt^, but not the PITPα^T59D^ mutant, which has diminished PI binding (Fig. S5, *A–D*). These results demonstrate that PITPα/β are necessary to generate nuclear PIP_n_ pools by a mechanism that requires PI binding.

### PITPα/β controls the PIP_n_ linkage to a large suite of cellular proteins

**Figure 3:**
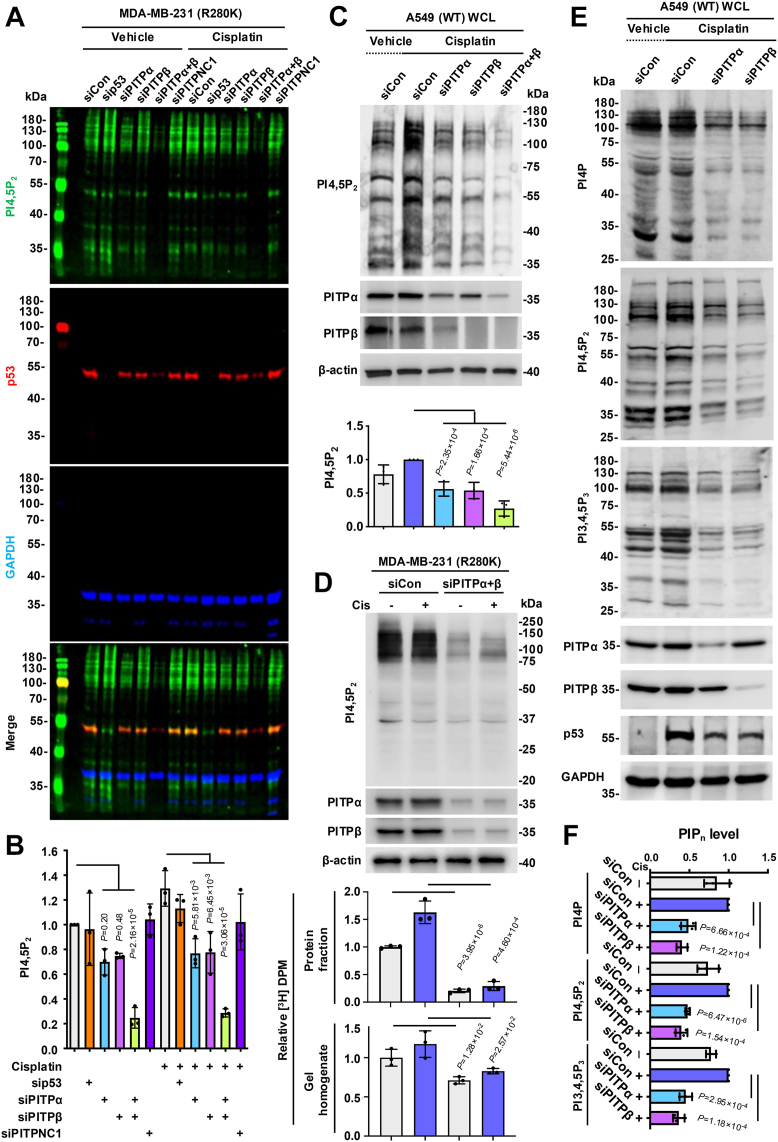
**PITPα/β are required for unique and stable protein-PIP_n_ complexes.***A* and *B*, MDA-MB-231 cells were transfected with control siRNAs or siRNAs against p53, PITPα, PITPβ, PITPNC1, or both PITPα and PITPβ. After 24 h, cells were treated with 30 μM cisplatin or vehicle for 24 h before being processed for triple fluorescent WB for detecting p53 (*red*), GAPDH (*blue*), and protein-bound PI4,5P_2_ (*green*) simultaneously. The protein-bound PI4,5P_2_ levels were quantified by ImageJ (*B*). n = 3 independent experiments. *p* value denotes ANOVA with Bonferroni's multiple comparisons test. *C*, A549 cells were transfected with control siRNAs or siRNAs against PITPα, PITPβ, or both PITPα and PITPβ. After 24 h, cells were treated with 30 μM cisplatin or vehicle for 24 h before being processed for WB. The protein-bound PI4,5P_2_ levels were quantified by ImageJ. n = 3 independent experiments. *p* value denotes ANOVA with Bonferroni's multiple comparisons test. *D*, MDA-MB-231 cells were cultured from low confluency in media containing in [^3^H]*myo*-inositol or unlabeled *myo*-inositol. After 48 h, cells were transfected with control siRNAs or siRNAs against PITPα and PITPβ. 24 h later, cells were treated with 30 μM cisplatin or vehicle for another 24 h. KD and loading were confirmed by WB before the protein fraction was extracted with CHCl_3_/MeOH (*top bar graph*) or resolved by SDS-PAGE, and the gel lane was excised (*bottom bar graph*). Extracted samples and dissolved gel sections were then analyzed by LSC. n = 3 independent experiments. *p* value denotes two-sided paired *t* test. *E* and *F*, A549 cells were transfected with control siRNAs or siRNAs against PITPα or PITPβ. After 24 h, cells were treated with 30 μM cisplatin or vehicle for 24 h before being processed for WB. The protein-bound PI4P, PI4,5P_2_ and PI3,4,5P_3_ levels were quantified by ImageJ (*F*). n = 3 independent experiments. *p* value denotes two-sided paired *t* test. For all graphs, data are represented as mean ± SD.

As PITPα/β regulate nuclear PIP_n_ levels and are required for p53-PIP_n_ complexes, we examined the potential role of PITPα/β in regulating PIP_n_ linkage to additional cellular proteins. We used PIP_n_ isomer-specific antibodies to immunoblot cell lysates by SDS-PAGE Western Blot (WB) to identify protein-PIP_n_ complexes in MDA-MB-231 breast cancer cells. Remarkably, WBs of whole cell lysates with a specific PI4,5P_2_ antibody detected many protein bands, including some that were enhanced by genotoxic stress (Fig. 3, *A* and *B*), indicating that PIP_n_s stably associate with many proteins. Moreover, individual and combined targeting of PITPα/β, both KD and KO models, robustly reduced the protein-coupled PI4,5P_2_ levels in response to stress (Figs. 3, *A* and *B* and S6*A*). Cisplatin treatment for 24 h achieved robust induction of protein-coupled PI4,5P_2_ levels (Fig. S6*B*). Similar results were obtained in A549 lung carcinoma cells which express wild type p53 (Fig. 3*C*). To further support the specificity of the PI4,5P_2_ antibody, we labeled cells with [^3^H]*myo*-inositol which is specifically incorporated into PI and then phosphoinositides and inositol phosphates (33). Consistent with the immunoblot findings, combined KD of PITPα/β also reduced [^3^H]*myo*-inositol labelling of total cellular proteins isolated by two complementary approaches, chloroform/methanol extraction followed by acetone precipitation, and analysis of proteins SDS-PAGE gels where the free PIP_n_ lipids migrate with the dye front (Fig. 3*D*). Furthermore, KD of PITPα/β reduced levels of multiple protein-bound PIP_n_s (PI4P, PI4,5P_2_, PI3,4,5P_3_) and many protein bands detected by PIP_n_-specific antibodies in whole cell lysates (Fig. 3, *E* and *F*), placing these PITPs as upstream regulators of PIP_n_ linkage to proteins.

### PI4KIIα cooperates with PITPα/β to synthesize protein-PIP_n_ complexes

**Figure 4:**
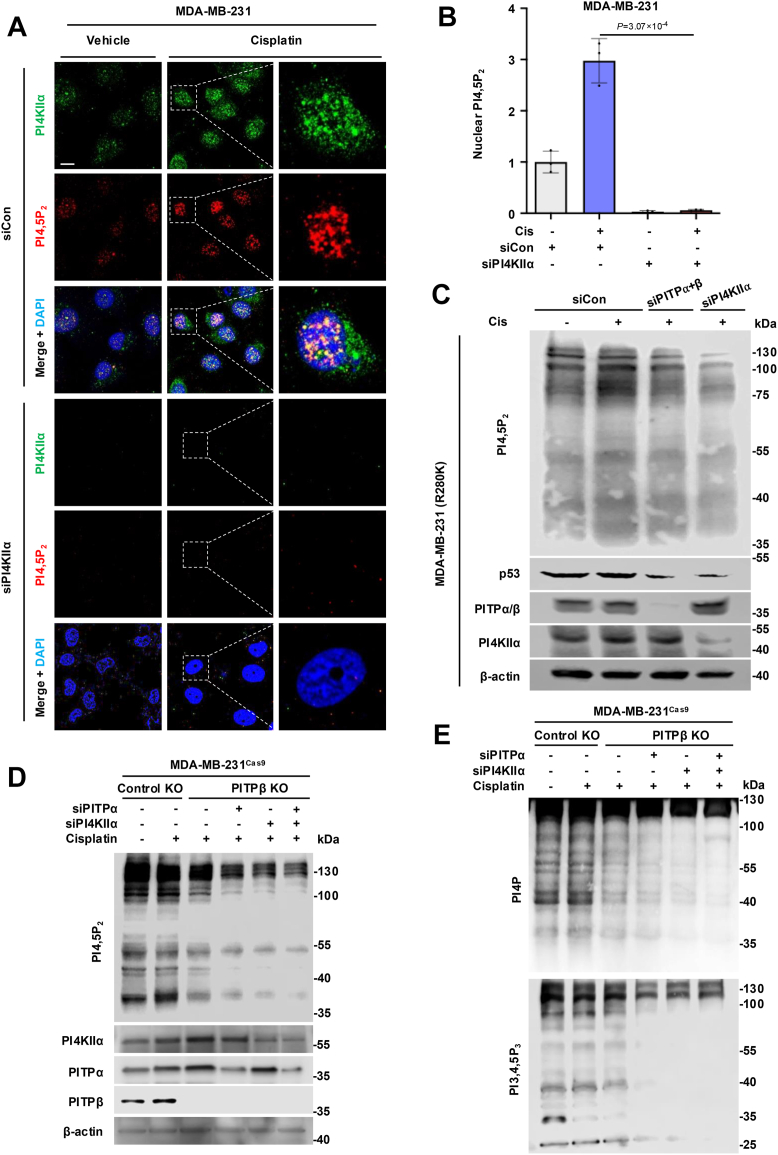
**PI4KIIα is required for nuclear and protein-bound PIP_n_ signaling.***A* and *B*, MDA-MB-231 cells were transfected with control siRNAs or siRNAs against PI4KIIα. After 24 h, cells were treated with 30 μM cisplatin or vehicle for 24 h before being processed for IF staining against PI4KIIα and PI4,5P_2_. PI4,5P_2_ levels were quantified using ImageJ (*D*). n = 3, average values were calculated from 15 cells from each independent experiment. *p* value denotes two-sided paired *t* test. *C*, MDA-MB-231 cells were transfected with control siRNAs or siRNAs against both PITPα and PITPβ or PI4KIIα. After 24 h, cells were treated with 30 μM cisplatin or vehicle for 24 h before being processed for WB. n = 3 independent experiments. *D* and *E*, MDA-MB-231^Cas9^ cells with PITPβ KO and control non-targeted KO were transfected with control siRNAs or siRNAs against PI4KIIα, PITPα or both PI4KIIα and PITPα. After 24 h, cells were treated with 30 μM cisplatin or vehicle for 24 h before being processed for WB against PI4,5P_2_ (*A*), PI4P (*B*), and PI3,4,5P_3_ (*B*). n = 3 independent experiments.For all graphs, data are presented as the mean ± SD. Scale bar, 5 μm.

As PITPα/β have been reported to cooperate with PI4Ks to synthesize PI4P (26, 34, 35) and p53-PIP_n_ complexes (see companion paper (22)), we postulated that PI4KIIα may also regulate synthesis of other protein-PIP_n_ complexes. Indeed, KD of PI4KIIα diminished basal and stress-induced nuclear PI4,5P_2_ levels (Fig. 4, *A* and *B*), similar to combined PITPα/β targeting. PI4KIIα KD also reduced protein-linked PI4,5P_2_ levels determined by WB (Fig. 4*C*). KD of PI4KIIα in the PITPβ KO MDA-MB-231^Cas9^ cells revealed that individual targeting of PITPα, PITPβ, or PI4KIIα is sufficient to inhibit protein-linked PIP_n_s but that combined targeting produces more robust inhibition (Fig. 4*D*). Similar results were observed for the other PIP_n_s known to be protein linked: PI4P and PI3,4,5P_3_ (Fig. 4*E*). These findings suggest that PITP-mediated PI cargo delivery, and subsequent generation of protein-linked PI4P by PI4KIIα, are required to enable subsequent PI4,5P_2_ and PI3,4,5P_3_ linkage.

### Proteomic analyses reveal potential pathways regulated by protein–PIP_n_ complexes

**Figure 5:**
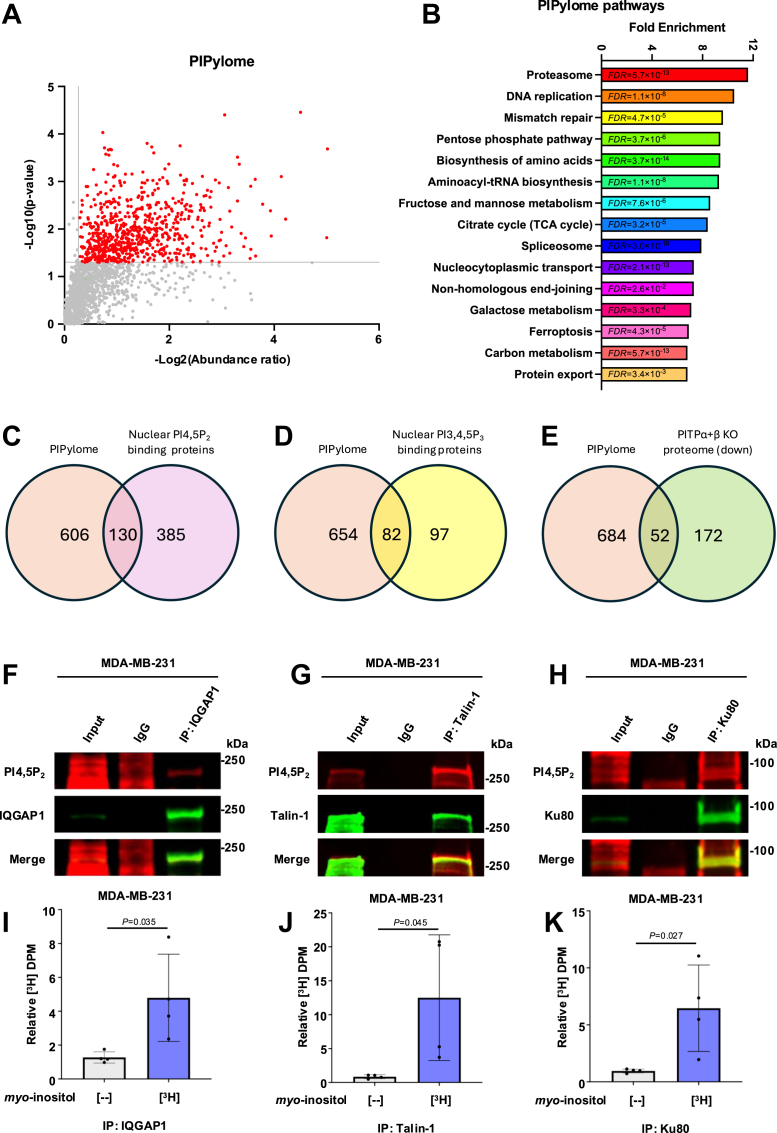
**Proteomic analysis of PITPα+β KO cells and PI4,5P_2_-linked proteins.***A* and *B*, MDA-MB-231 cells were cultured under normal conditions and lysed. Then proteins were precipitated using chloroform/methanol, resuspended and incubated with anti-PI4,5P_2_ antibody. The antibody and associated proteins were then recovered using Dynabeads and submitted for mass spectrometry proteomic analysis. Identified proteins were then graphed (*A*) and submitted for pathway enrichment analysis using ShinyGO 0.85 (*B*). T-tests were used to assess significance and fold change of 0.8 to 1.2 were used as cut offs. *C–E*, PI4,5P_2_-linked proteins (PIPylome) were compared against an available nuclear PI4,5P_2_ interactome (*C*) (36), nuclear PI3,4,5P_3_ interactome (*D*) (37), and downregulated proteins in MDA-MB-231 PITPα+β KO cells (*E*). *F–H*, MDA-MB-231 cells were cultured and lysed under normal conditions and IQGAP1 (*F*), Talin-1 (*G*), and Ku80 (*H*) were immunoprecipitated and resolved *via* WB along with PI4,5P_2_. n = 3 independent experiments. *I–K*, MDA-MB-231 cells were cultured in media containing in [^3^H]*myo*-inositol or unlabeled *myo*-inositol. Cells were lysed and IQGAP1 (*I*), Talin-1 (*J*), and Ku80 (*K*) were immunoprecipitated and resolved *via* SDS-PAGE. Corresponding protein bands were then excised from the gel and dissolved before being analyzed by LSC. n = 4 independent experiments. For all graphs, data are presented as the mean ± SD.

Given the observation that many cellular proteins form stable protein-PIP_n_ linkages, we set out to define this subset of lipid-regulated proteins. First, we developed an IP-Mass Spectrometry approach using the anti-PI4,5P_2_ antibody to identify proteins that are PI4,5P_2_-linked (“PIPylome”). Total cellular proteins were precipitated from cell lysates with chloroform/methanol to reduce competing PI4,5P_2_ lipid, dissolved in buffered SDS, diluted and combined with anti-PI4,5P_2_ antibody. The complexes were isolated by a Dyna-bead approach to enrich for PI4,5P_2_-linked proteins, eluted and analyzed by mass spectrometry (Fig. S7*A*). Many proteins were identified that specifically bind the anti-PI4,5P_2_ antibody, including several that were already known to form these stable protein-PIP_n_ complexes and others that were first identified by this approach (Fig. 5*A* and Table S1). These proteins regulate many cellular processes including DNA damage response pathways (*e.g*., DNA replication, mismatch repair and non-homologous end-joining), metabolism (*e.g*., TCA cycle, pentose phosphate pathway, carbon and galactose), ferroptosis, signaling, regulators of cytoskeletal dynamics and motility (Fig. 5*B*).

We then built on the observation that for p53, as well as Star-PAP and NRF2, PIP_n_ linkages result in increased protein stability *via* recruitment of the small heat shock proteins αB-crystallin and HSP27 (14, 20, 21). We successfully created double PITPα and PITPβ KO MDA-MB-231^Cas9^ cells, effectively eliminating the class I PITPs from this cell line (22). We then submitted lysates for proteomic analysis compared to a control KO in MDA-MB-231^Cas9^ cells to identify downregulated proteins in PITPα+β KO cells (Table S2). Multiple downregulated proteins were identified in PITPα+β KO cells using this method, including PITPβ and p53 as internal controls (Fig. S7*B*). Gene set enrichment analysis revealed key proteins involved in cancer metabolism and progression were altered in PITPα+β KO cells (Fig. S7, *C* and *D*).

To further distinguish PIP_n_-linked proteins from PIP_n_ binding proteins, we compared our PIPylome to available PIP_n_ interactomes. Interestingly, the PIPylome has 130 proteins in common with a nuclear PI4,5P_2_ interactome (∼25%) (36) and 82 proteins in common with a nuclear PI3,4,5P_3_ interactome (∼46%) (37), suggesting many proteins exhibit both types of PIP_n_ interaction frameworks (Fig. 5, *C* and *D* and Table S3). Moreover, the PIPylome has 52 proteins (∼23%) that were also downregulated in PITPα+β KO cells, indicating both shared and divergent regulatory mechanisms with the established p53 and Star-PAP pathways (Fig. 5*E* and Table S3) (14, 20, 21). To validate the anti-PI4,5P_2_ affinity purification followed by MS analysis, we selected three proteins that were enriched in the PIPylome. IQGAP1, Talin-1, and Ku80 all when IP’ed retained linked PI4,5P_2_ by Western blotting and [^3^H]*myo*-inositol labeling, confirming that this IP-MS approach identifies PI4,5P_2_-linked proteins (Fig. 5, *F–K*). Taken together, these data highlight a subset of proteins that form unique and stable complexes with PIP_n_, that are involved in fundamental cellular processes (Fig. 5*B*).

## Discussion

**Figure 6:**
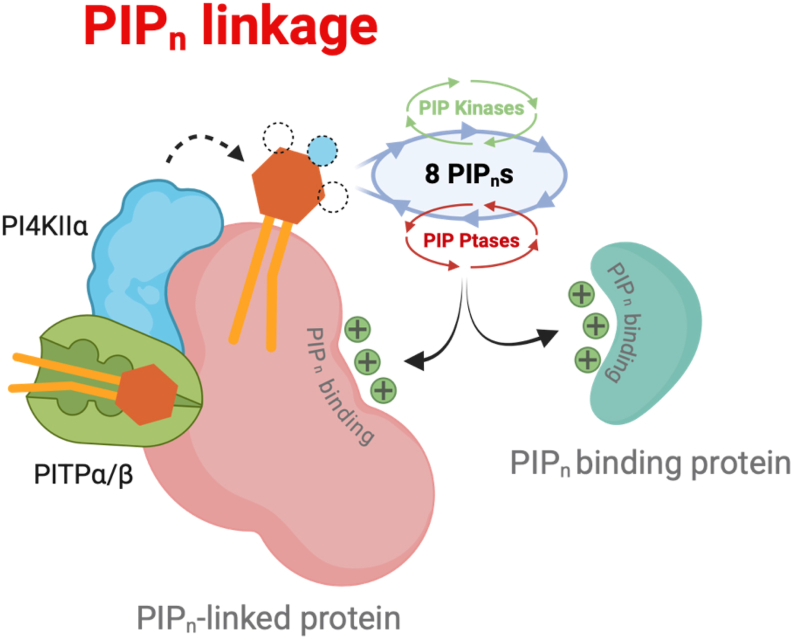
**Emerging model of the PIPylation pathway.** The data indicate that proteins which will become PIPylated interact with PITPα/β and the PI4KIIα. This results in the generation of a protein-PI4P complex that is well defined in the case of p53 (see companion paper) (22). The protein–PI4P complexes are then acted on by PIP kinases and/or PIP phosphatases (PIP Ptases) to generate eight potential PIP_n_ linked complexes including PI (PI3P, PI4P, PI5P, PI3,4P_2_, PI4,5P_2_, PI3,5P_2_, and PI3,4,5P_3_). As many of the PIP_n_-linked proteins are also PIP_n_-binding proteins, the linked PIP_n_ recruits other PIP_n_ binding proteins and/or may bind intramolecularly to itself. This is the case with p53–PIP_3_ complex that recruits Akt pathway components all of which specifically bind PI3,4,5P_3_.

In the companion paper, we discovered that the PI transfer proteins PITPα/β bind wild-type and mutant p53, and together with the PI 4-kinase, PI4KIIα, generate p53-PI4P that is required for the synthesis of other p53-PIP_n_ complexes (22). The linked PIP_n_ isomers regulate p53 stability and function (13, 14). Moreover, PIP_n_s are linked to other proteins, including Star-PAP, NRF2, YAP/TAZ and MDM2 (16, 19, 20, 21). Here we demonstrate that PITPs and PI4KIIα play a broader role in linking PIP_n_s to other proteins. Remarkably, WB with antibodies specific for PIP_n_ isomers revealed many proteins have stably linked PIP_n_s, a fundamentally distinct mechanism from conventional PIP_n_ binding. Metabolic labeling of cells with the PIP_n_ precursor [^3^H]-*myo*-inositol resulted in the stable incorporation of [^3^H] label into protein that withstands boiling and SDS-PAGE, further underscoring that the PIP_n_s are protein linked. Moreover, both the protein bands identified by WB with PIP_n_ antibodies and by [^3^H]-*myo*-inositol incorporation are reduced by individual KD/KO of PITPα/β, while combined KD/KO of PITPα/β resulted in more robust suppression. PI binding by PITPα is required to rescue the effects of PITPα/β KD/KO, highlighting that this well-described function of these transfer proteins is required to initiate PIP_n_ linkage to proteins. PI4KIIα KD phenocopies PITPα/β KD/KO, indicating that both are necessary for PIP_n_-linkage to proteins. Consistent with this new function of PITPs, their cellular localization responds dynamically to diverse cellular stressors, resulting in their accumulation in the nucleus corresponding to generation of a PIP_n_ pool, which is anchored to proteins. These data point to a unique panel of PIP_n_-linked proteins, the “PIPylome”, that requires the combined actions of PITPα/β and PI4KIIα (Fig. 6). We are actively investigating the chemical nature of this putative posttranslational modification and the amino acid(s) involved in this linkage.

While several lines of evidence support the accuracy and specificity of the PIP_n_ antibodies (13, 14, 16, 20, 22, 29, 38, 39, 40), it is important to note that their application in this manuscript is does have potential limitations. Namely, the removal of the intended lipid tagets by detergent, SDS-PAGE, or protein precipitation may increase the possibility of non-specific interactions. Additionally, some antibodies require unique optimizations for their successful application in immunoprecipitation workflows as we have done to define the PIPylome (41). Especially in the context of a modification-targeted antibody, one can imagine steric hinderance or other biochemical considerations could limit the complete enrichment of proteins with a PI4,5P_2_ linkage. The PIPylome defined by PI4,5P_2_-linked proteins was further characterized by comparing it to three other potentially related proteomes: (1) a proteome of PI,4,5P_2_-binding proteins; (2) a proteome of PI3,4,5P_3_-binding proteins; and (3) a proteome in control *versus* combined PITPα/β KO cells. All of the PIP_n_-linked proteins that have been analyzed to date also bind PIP_n_ isomers with high affinity, resulting in their regulation (16, 19, 20, 21) (Fig. 6). This suggests that the linked PIP_n_ may bind intramolecularly, consistent with our observation that a subset of PIPylome proteins are present in the PI4,5P_2_ and PI3,4,5P_3_ interactomes of nuclear proteins. Furthermore, PIP_n_ linkage regulates the stability of PIPylated proteins, including p53, MDM2, Star-PAP, and NRF2, by recruiting small heat shock proteins (13, 14, 16, 19, 20, 21). Pathway analysis identified proteins of several pathways that are differentially expressed in the presence and absence of PITPα/β, a subset of which are also concordantly enhanced in the PIPylome (Fig. 5*B*). Select proteins in the PIPylome are discussed to illustrate the significance and links to both cellular pathways and phosphoinositide signaling.

Ku80 is a protein in the PIPylome, and present in the PI4,5P_2_ and PI3,4,5P_3_ interactomes, for which we validated the PIP_n_ linkage (Fig. 5*H*). Ku80 and Ku70 (also present in the PIPylome and PI3,4,5P_3_ interactome) form a heterodimer to recruit DNA-PK to sites of DNA damage (42, 43). Remarkably, PARP1 is also in the PIPylome and PI3,4,5P_3_ interactome, and it binds and regulates Ku80 and Ku70, suggesting a functional role of PIPylation in the DNA damage response, consistent with prior reports (13, 14, 22, 39, 44, 45). Indeed, DNA damage induces the rapid accumulation of PIP_n_s to sites of DNA damage where they regulate DNA-PK and ATR (39, 45) and p53 (13, 14) (all PIP_n_-linked proteins) (45) in the DNA damage response. The observation that multiple DNA damage pathway components are in the PIPylome and highlighted by pathway analysis, strongly suggests an important functional role for PIPylation in this process.

Although the role of the PITPs and PI4KIIα in PIP_n_ linkage to nuclear proteins is supported by the robust decrease in PI4,5P_2_ nuclear staining upon PITP and/or PI4KIIα KD, there is also a clear decrease in cytosolic PIP_n_ staining by PITP and/or PI4KIIα KD. Indeed, many cytoplasmic proteins are identified in the PIPylome. We validated two cytoplasmic proteins in the PIPylome (IQGAP1 and talin). IQGAP1 is a cytoplasmic protein that binds PI4,5P_2_ and also scaffolds the full agonist-stimulated membrane-localized PI3K/Akt pathway and regulates cell migration and proliferation (6, 46). These data suggest that PIP_n_ linkage to IQGAP1 may regulate the assembly and/or activation of canonical PI3K/Akt signaling, cell migration and/or other functions. Interestingly, IQGAP1 has also been identified in the nucleus (47), is present in the nuclear PI3,4,5P_3_ interactome, and is a known PI3,4,5P_3_ binding protein (48), suggesting a potential nuclear function as well. The cytoplasmic protein talin directly binds PI4,5P_2_, which regulates its interaction with integrins and the PI4,5P_2_-generating PIP kinase PIPKIγi2 to control cell adhesion, migration, invasion and epithelial-mesenchymal transition (49, 50, 51). These data implicate PIP_n_-linked talin in these functions that promote tumor progression and metastasis. Consistent with our model (Fig. 6), PIP_n_s both bind and are stably linked to IQGAP1 (6, 46) and talin (49, 50, 51), suggesting that the linked PIP_n_s regulate inter- and intramolecular interactions. Taken together, our findings indicate that PIPylation is not limited to nuclear proteins or any specific cellular compartment.

PIPylation is also positioned to have a functionally important role in cell division. The cytoskeletal septins-7, -9, and -11 are all present in the PIPylome and the PI3,4,5P_3_ interactome. Septin-9 has polybasic domains that binds PI4P and are critical for its role in filament assembly (52). The protein regulator of cytokinesis 1, PRC1, is also in the PIPylome but has not been identified as a PIP_n_-binding protein. PRC1 is a microtubule-associated protein essential for the final stages of cell division by stabilizing the midzone microtubule bundle, permitting completion of cell cleavage (53). PIPylation of the septins and PRC1 is consistent with the roles of the PIP_n_s in the later stages of cell division as changes in PI4,5P_2_ levels at the cleavage furrow are essential for completion of cytokinesis (54, 55, 56). Further, local PI4,5P_2_ synthesis by septin binding the PIPKIγi3/i5 C-terminal isoform (57, 58) control the centralspindlin association with the midbody during cytokinesis (56). Clearly, these PIP_n_ linkages will need to be validated, and functional studies performed to precisely define the role of PIPylation in these events, but the discovery of the PIP_n_-linked proteome provides a new lens to generate testable hypotheses.

In summary, we have provided evidence that the PIP_n_ isomers (PI4P, PI4,5P_2_ and PI3,4,5P_3_) are stably linked to proteins in the nucleus and cytoplasm. There are potentially more than 700 putative PIP_n_-linked proteins and given the existence of seven distinct PIP_n_ isomers (4, 5), it seems plausible that additional PIP_n_ isomers are also linked to proteins to modulate their signaling functions (Fig. 6). Notably, the protein-linked PIP_n_s are signaling competent as they are modified by specific PIP kinases and phosphatases and each isomer selectively recruits a set of downstream effectors, *e.g*., small heat shock proteins, Akt-activating kinases and Akt substrates (4, 5, 13, 14, 19, 20, 21). Significantly, the interactions of p53 with these PIP metabolizing enzymes and Akt pathway components occurs in the absence of the PIP_n_ linkage (with *E*. *coli* expressed p53) (13, 14). This indicates that in the absence of PIP_n_ linkages, protein-protein interactions provide the functional specificity for p53 and likely other proteins, but the PIP_n_-linkage further controls the recruitment and activity of specific protein effectors of the PIP_n_-linked protein(s) (16, 19, 20, 21). Thus, the linkage of PIP_n_ second messengers to proteins represent a “third messenger” pathway that is positioned to function independently of the canonical membrane-localized pathway, fine tuning the interactions and functions of the linked protein, the details of which are just beginning to emerge.

## Experimental procedures

### Cell culture and constructs

All cell lines including A549, BT-549, Cal33, HCT116, HS578T, MDA-MB-231, SUM159, SUM1315, HEK293FT, and MCF-10A cells were purchased from ATCC. MCF10A cells were grown in DMEM/F12 (#11330-032, Invitrogen) with supplements of 10% fetal bovine serum (#100–106, GeminiBio), 1% penicillin/streptomycin (#15140-122, Gibco), 20 ng/ml EGF (#CC-4107, Lonza), 0.5 mg/ml Hydrocortisone (#H4001, Sigma), 100 ng/ml Cholera toxin (#C-8052, Sigma), and 10 μg/ml Insulin (#I-1882, Sigma). The remaining cell lines were maintained in DMEM (#10-013-CV, Corning) supplemented with 10% fetal bovine serum (#100–106, GeminiBio) and 1% penicillin/streptomycin (#15140-122, Gibco). The cell lines used in this study were routinely tested for *mycoplasma* using MycoAlert Kit (#LT07-318, Lonza), and mycoplasma-negative cells were used. None of the cell lines used in this study are listed in the database of commonly misidentified cell lines maintained by ICLAC. The HA-tagged wild-type PITPα construct and PI-binding deficient mutant T59D (59) were purchased from Genscript. Plasmids were transfected according to the manufacturer’s instructions into mammalian cells using Lipofectamine3000, (#L3000015, Thermo Fisher Scientific). Typically, 2 to 5 μg of DNA was used alongside 6 to 10 μl of lipid in 6-well plates for transfection. Cells were selected for at least 80% transfection efficiency and used for further analysis.

### Antibodies and reagents

The monoclonal antibodies that were used are against p53 (clone DO-1, #SC-126, Santa Cruz Biotechnology), p53 (clone 7F5, #2527, Cell Signaling), pAkt^S473^ (clone 193H12, #4058, Cell Signaling), HA-tag (clone C29F4, #3724, Cell Signaling), GAPDH (clone 0411, #sc-47724, Santa Cruz Biotechnology), β-actin (clone 13E5, #4970, Cell Signaling), Lamin B2 (clone D8P3U, #12255, Cell Signaling), IQGAP1 (clone D-3, #sc-374307, Santa Cruz Biotechnology), Talin-1 (clone C-9, #sc-365875, Santa Cruz Biotechnology), Ku80 (clone C48E7, #2180, Cell Signaling), and polyclonal antibodies against PITPα (#16613-1-AP, ThermoFisher), PITPβ (#ab127563, abcam), PITPNC1 (IF, #ab222078, abcam), PITPNC1 (WB, #NBP2-19842, Novus), PI4KIIα (#NBP2-44158, Novus). Anti-PI4P (#Z-P004, Echelon), PI4,5P_2_ (#Z-P045, Echelon) and PI3,4,5P_3_ (#Z-P345, Echelon) antibodies were used for immunostaining, proximity ligation assay (PLA), and WB analyses. For immunoblotting analyses, all antibodies were diluted at a 1:1000 ratio except for GAPDH (clone 0411, 1:5000) and p53 p53 (clone DO-1, 1:5000). For protein immunoprecipitation, antibody-conjugated agarose was purchased from Santa Cruz Biotechnology, including agarose-conjugated antibodies against IQGAP1 (#sc-374307AC), Talin-1 (#sc-365875AC), and Ku80 (#sc-5280AC). For immunostaining analyses and proximity ligation assay PLA, all primary antibodies were diluted at a 1:100 ratio. Nuclear envelope marker (Alexa Fluor488 Lamin A/C, clone 4C11, #8617, Cell Signaling, 1:200) was used to identify the nuclear boundry. For the knockdown (KD) experiments, the ON-TARGETplus siRNA SMARTpool with 4 siRNAs in combination against human PITPα (#L-018010–00), PITPβ (#L-006459–01), PITPNC1 (#L-012476–02), and PI4KIIα (#LQ-006770–00–0020) were purchased from Dharmacon. Non-targeting siRNA (#D-001810–01, Dharmacon) was used as a control. For the KD and rescue experiments, methodology was the same as in the companion manuscript for this publication (22) and KD efficiency was determined by immunoblotting. KD efficiency greater than 80% was required to observe phenotypic changes in the study. Cisplatin (#NC1706394, Fisher Scientific) was used as cellular stressors.

### Immunoprecipitation and immunoblotting

Immunoprecipitation methodology was performed the same as found and described in the companion manuscript for this publication (22). Immunoblots were developed by Odyssey Imaging System (LI-COR Biosciences) and protein levels were quantified using ImageJ. The unsaturated exposure of immunoblot images was used for quantification with the appropriate loading controls as standards. Statistical data analysis consisted of *t* test and ANOVA and was performed with Microsoft Excel and PRISM, respectivley, using data from at least three independent experiments.

### Fluorescent IP-WB

Cells were lysed in a RIPA lysis buffer system (13) after the indicated treatment and quantified for protein concentration as described above. For endogenous protein immunoprecipitation, 0.5 to 1 mg of cell lysates were incubated with 20 μl anti-IQGAP1 (clone D-3, #sc-374307AC, Santa Cruz Biotechnology), Talin-1 (clone C-9, #sc-365875AC, Santa Cruz Biotechnology), or Ku80 (clone B-1, #sc-5280AC, Santa Cruz Biotechnology) monoclonal IgG antibody-conjugated agarose at 4 °C for 24 h. Normal immunoglobulin (IgG)-conjugated agarose was used as a negative control (#sc-2343, Santa Cruz Biotechnology). IP methodology is the same as described in the companion manuscript for this publication (22). For double fluorescent IP-WB detecting protein-PI4,5P_2_ complexes, anti-IQGAP1 (#sc-374307), -Talin-1 (#sc-365875), or -Ku80 (#2180) rabbit monoclonal IgG antibody at 1:2000 dilution and anti-PI4,5P_2_ mouse monoclonal IgM antibody (#Z-P045, Echelon) at 1:1000 dilution were mixed together in blocking buffer with 0.02% Sodium Azide and incubated with the membrane at 4 °C overnight. The next day, the membrane was thrice washed with TBST for 10 min. Secondary antibody incubation and imaging were conducted as described in the companion manuscript (22). Statistical data analysis was performed with Microsoft Excel, using data from at least three independent experiments.

### Immunofluorescence and confocal microscopy

For immunofluorescence studies, methodology, statistical testing, quantification and correlation analysis was conducted as described in the companion manuscript (22). A value of 1 represents perfect correlation, 0 means no correlation, and −1 means perfect negative correlation (60). Pearson’s *r* >0.7 suggests a strong correlation (61).

### [^3^H]*myo*-inositol metabolic labeling

Radio-labeling experiments were conducted as described in the companion manuscript for this publication (22).

### Lenti-CRISPR gene editing

All gene editing was conducted as described in the companion manuscript for this publication (22).

### Mass spectrometry proteomics of MDA-MB-231 PITPα+β KO cells

MDA-MB-231 PITPα+β KO cells were generated as described above in *Lenti-CRISPR Gene Editing*. Parental and KO cells were plated in triplicate and grown to ∼80% confluency after 24 h. The plates were then lysed using RIPA and batched together to minimize variability before MeOH:CHCl_3_ (2:1) was added to form protein precipitate. Soluble phases in MeOH and CHCl_3_ were aspirated out and the protein flake was dried using a speed vacuum. Pellets were resolubilized into 500 μl 8M urea (#U15, Fisher) in 50 mM ammonium bicarbonate (#A643, Fisher), pH 8.5. 25 μl of this was taken and diluted to 4M urea with 50 mM ammonium bicarbonate. Samples were reduced with 2 mM TCEP (#C4706, Sigma) for 45 min at 42 C, then alkylated with 5 mM iodoacetamide (#I1149, Sigma) for 45 min in the dark at room temperature, then quenched with another aliquot of 2 mM TCEP for 5 min. Samples were diluted to 1M urea with 50 mM ammonium bicarbonate and proteolytically digested for 12 h at 37 °C using a 1:1 mixture of Trypsin:Lys-C (#V5071, Promega) and a protease:protein ratio of 1:50. Samples were acidified by addition of neat formic acid (#A117, Fisher) to 1% vol/vol, desalted and concentrated using OMIX C18 tips (#A57003100, Agilent) and manufacturer’s protocol, and dried to completion with a vacuum centrifuge. Resulting dried down peptides were resolubilized into 50 μl 0.1% formic acid (#LS118, Fisher) and 3 μM of this was injected for spectral analysis.

The liquid chromatography (LC) system used was a Dionex UltiMate 3000 and the LC conditions were as follows: buffer A is 0.1% formic acid (#LS118, Fisher), buffer B is 80/20/0.1% acetonitrile/water/formic acid, flow rate was 300 nl/min, and the column used was a 50 cm Thermo Scientific PepMap RSLC C18 column with 2 μm bead size, 100 Å pore size, and 75 μm inner diameter. After column loading in 2% B, peptide samples were eluted with a 75-min linear gradient from 0 to 25%B, followed by a 20-min linear gradient from 25 to 50%B, a 4 min linear gradient to 95%B, flushing with 05% B for 3 min, then requilibration to 2% B for 15 min. MS1 acquisition conditions were as follows: cycle time of 1s between MS1 scans, acquisition in the orbitrap mass analyzer with a resolution of 120K, scan range of 350-1600 m/z, normalized AGC target of 250%, profile data and positive mode, and a max inject time of 50 ms. Fragment ion selection was filtered using MIPS mode set to peptide and charge state set to 2 to 6. Dynamic exclusion was used with an n of 1 for 10 s, and a mass tolerance ± 10 ppm. MS2 spectra were acquired in the ion trap with quadrupole isolation of 0.7, HCD activation/fragmentation with 30% collision energy, turbo scan rate, max inject time of 25 ms, normalized AGC target of 300%, scan range set to auto, and positive mode with centroid data. Raw data was searched with the software Proteome Discoverer v2.4. The H. Sapiens Uniprot proteome (20353 sequences) was searched using the Sequest algorithm alongside a database of common contaminants with the following parameters: a precursor mass tolerance of 10 ppm, fragment ion mass tolerance of 0.6 Da, max missed cleavages of 2, carbamidomethylation (+57.02) set as a fixed modification on cysteines. The following modifications were set as dynamic: oxidation (+15.99) on methionines, deamidation (+0.98) on asparagines and glutamines, and phosphorylation on serines, threonines, and tyrosines (+79.97). A concatenated database search strategy was used in the Percolator node, and an FDR of 0.05 was set. For scoring and further filtering, all default parameters were used. For label-free quantification in Proteome Discoverer, peptide precursor abundances based on intensity and summed abundances were used. Modified peptides were not included in quantification.

### Mass spectrometry proteomics of anti-PI4,5P_2_ immunoprecipitation

MDA-MB-231 cells were plated in triplicate and allowed to reach ∼80% confluency. The cells were then lysed using a RIPA lysis system (#sc-24948, Santa Cruz Biotechnology) with 1 mM Na3VO4, 5 mM NaF, and 1x protease inhibitor cocktail (#11836153001, Roche). The triplicate groups were then compiled into a single batched lysate, and proteins were precipitated using a 4:1 ratio of Chloroform:Methanol. The protein precipitate was resuspended in 3% SDS in PBS and split into 6 tubes; 3 tubes were incubated with 10 μg anti-PI4,5P2 IgM antibody (#Z-P045, Echelon) and 3 tubes were incubated with normal IgM antibody (sc-3881, Santa Cruz) for 24 h while rotating. After 24 h, the resuspended proteins were added to pre-cleared anti-mouse IgM Dynabeads (#11039D, Invitrogen) and rotated at room temperature for 1.5 h. The bound complexes were then purified *via* magnet and bound proteins were eluted using 0.1 M citrate. Eluates were brought to 5% final SDS. Samples were reduced for 35 min with 2 mM Tris(2-carboxyethyl)phosphine hydrochloride (TCEP, Sigma, #C4706) at 42 °C, alkylated for 35 min with iodoacetamide (#I1149, Sigma) at room temperature in the dark, then quenched with a further 2 mM TCEP for 5 min at room temperature. Samples were digested on S-Trap Micro digestion columns (Protifi) per manufacturer’s protocol with 3 μg of Trypsin/Lys-C mix (#V5071, Promega). Resulting dried down peptides were resolubilized into 10 μl 0.1% formic acid (#LS118, Fisher) and 1.5 μl of this was injected for spectral analysis as described above in Mass Spectrometry proteomics of MDA-MB-231 PITPα+β KO cells.

Samples were pooled prior to pulldown with the specified antibodies, but replicate pulldowns were performed to specifically assess reproducibility and statistical significance of proteins bound by anti-PI4,5P_2_ antibody vs. the IgM background independent of any biological variability. T-testing was performed between the triplicate calculated abundances of proteins that were pulled down by the anti-PIP2 antibody vs. IgM alone.

### Statistics and reproducibility

One-way ANOVA was used for group significance with Bonferroni's multiple comparisons test used for significance within grouped samples and Two-tailed unpaired *t*-tests were used for pair-wise significance. In this study, no power calculations were used. Experimental design and sample sizes were determined based on previously published experiments where significance was readily observed (13, 14). As noted, each experiment was independently repeated at least three times, and the number of repeats is defined in the figure legend. We used at least three independent experiments or biologically independent samples for statistical analysis.

## Data availability

All data supporting the findings of this study are available from the corresponding authors on reasonable request.

## Supporting information

This article contains supporting information.

## Conflict of interest

The authors declare that they have no conflicts of interest with the contents of this article.
