# Supplementary material for: Lipid transfer proteins and PI4KIIα generate a phosphoinositide-linked proteome
Source: J Biol Chem. 2026 May 5;302(6):113089. doi: 10.1016/j.jbc.2026.113089 (PMC13266111; doi:10.1016/j.jbc.2026.113089)
Supplement: Supplemental figures [file mmc4.pdf]

Extended Fig. 1

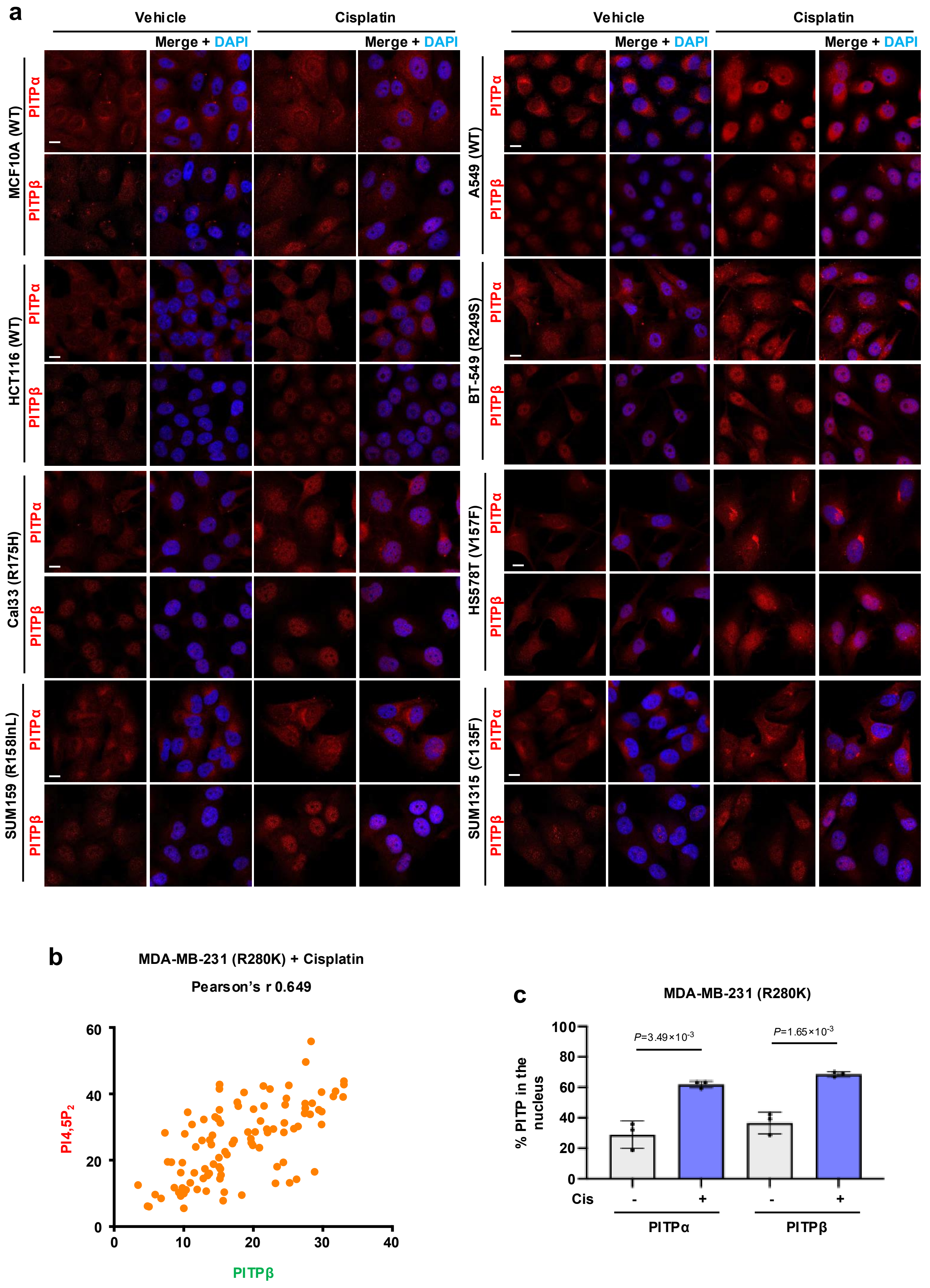

Extended Data Figure 1. PITPα/β nuclear accumulation in multiple cell lines

a, Confocal IF of PITPα or PITPβ in MCF10A (WT), A549 (WT), HCT116 (WT), BT-549 (R249S), Cal33 (R175H), HS578T (V157F), SUM159 (R158InL), and SUM1315 (C135F) cells treated with vehicle or 30 μM cisplatin for 24 h. The nuclei were counterstained by DAPI. See quantification in Fig. 1c. n=3 independent experiments.

b, Correlation analysis using LASX between PITPβ and $PI4,5P_2$ levels as determined by IF in MDA-MB-231 cells treated with 30 μM cisplatin for 24 h. Pearson's r=0.649.

c, Localization analysis of PITPα/β as determined by IF in MDA-MB-231 cells treated with 30 μM cisplatin for 24 h. Nuclear regions were defined by DAPI counterstaining and nuclear IF signals were divided by total IF signals for individual cells. n=3, average values were calculated from 7 cells from each independent experiment. p value denotes two-sided paired t-test.

For all graphs, data are presented as the mean ± SD. Scale bar, 5 μm.

Extended Fig. 2

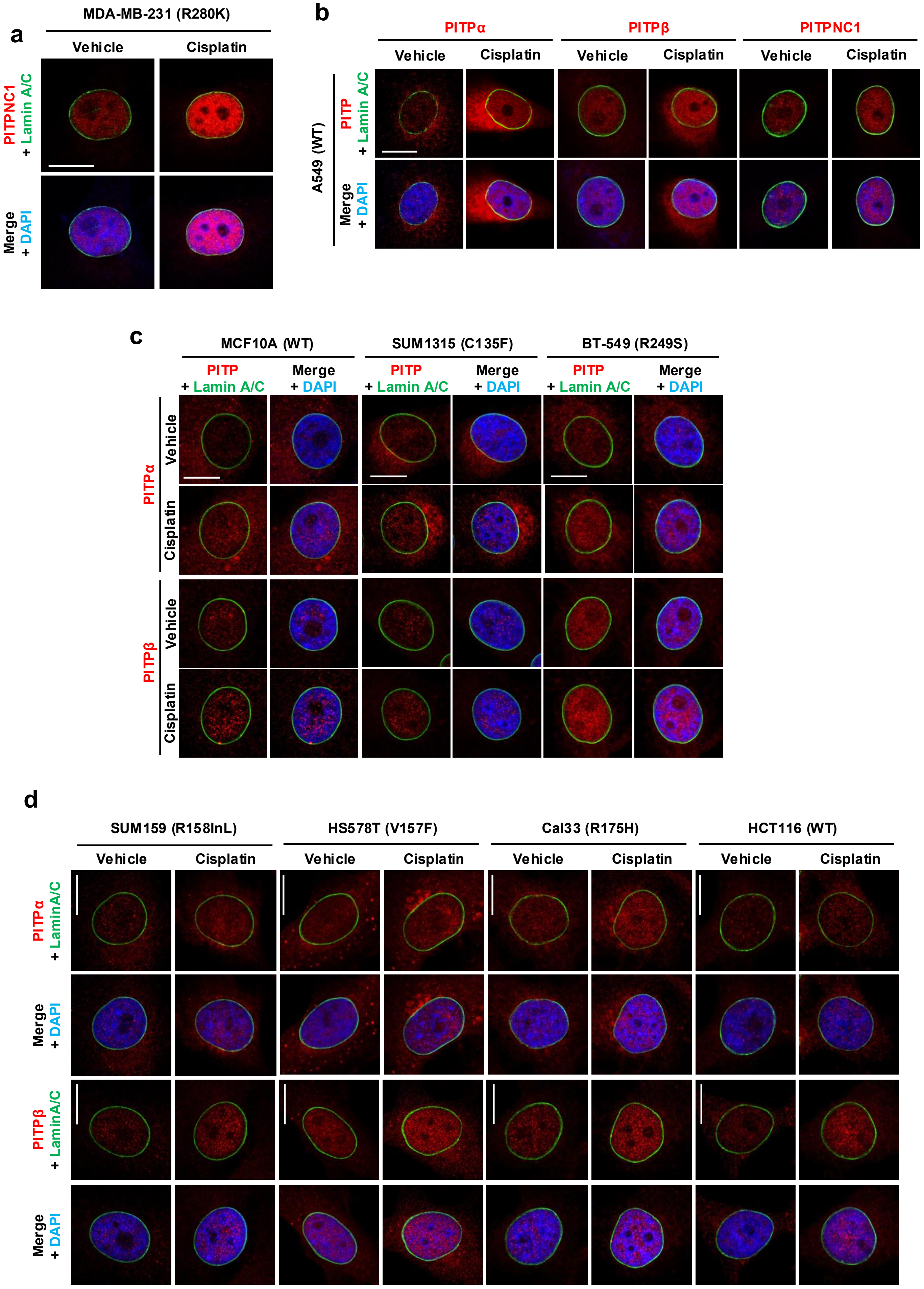

Extended Data Figure 2. PITPα/β accumulate in the non-membranous nucleoplasm

a, Confocal IF of PITPNC1 overlaid with the nuclear envelope marker Lamin A/C in MDA-MB-231 cells treated with vehicle or 30 μM cisplatin for 24 h. The nuclei were counterstained by DAPI. See PITPα and PITPβ IF in Fig. 1d. n=3 independent experiments.

b, Confocal IF of PITPα, PITPβ, or PITPNC1 overlaid with the nuclear envelope marker Lamin A/C in A549 cells treated with vehicle or 30 μM cisplatin for 24 h. The nuclei were counterstained by DAPI. n=3 independent experiments.

c, Confocal IF of PITPα or PITPβ overlaid with the nuclear envelope marker Lamin A/C in MCF10A (WT), SUM1315 (C135F), and BT-529 (R249S) cells treated with vehicle or 30 μM cisplatin for 24 h. The nuclei were counterstained by DAPI. n=3 independent experiments.

d, Confocal IF of PITPα or PITPβ overlaid with the nuclear envelope marker Lamin A/C in SUM159 (R158InL), HS578T (V157F), Cal33 (R175H), and HCT116 (WT) cells treated with vehicle or 30 μM cisplatin for 24 h. The nuclei were counterstained by DAPI. n=3 independent experiments.

Scale bar, 5 μm.

Extended Fig. 3

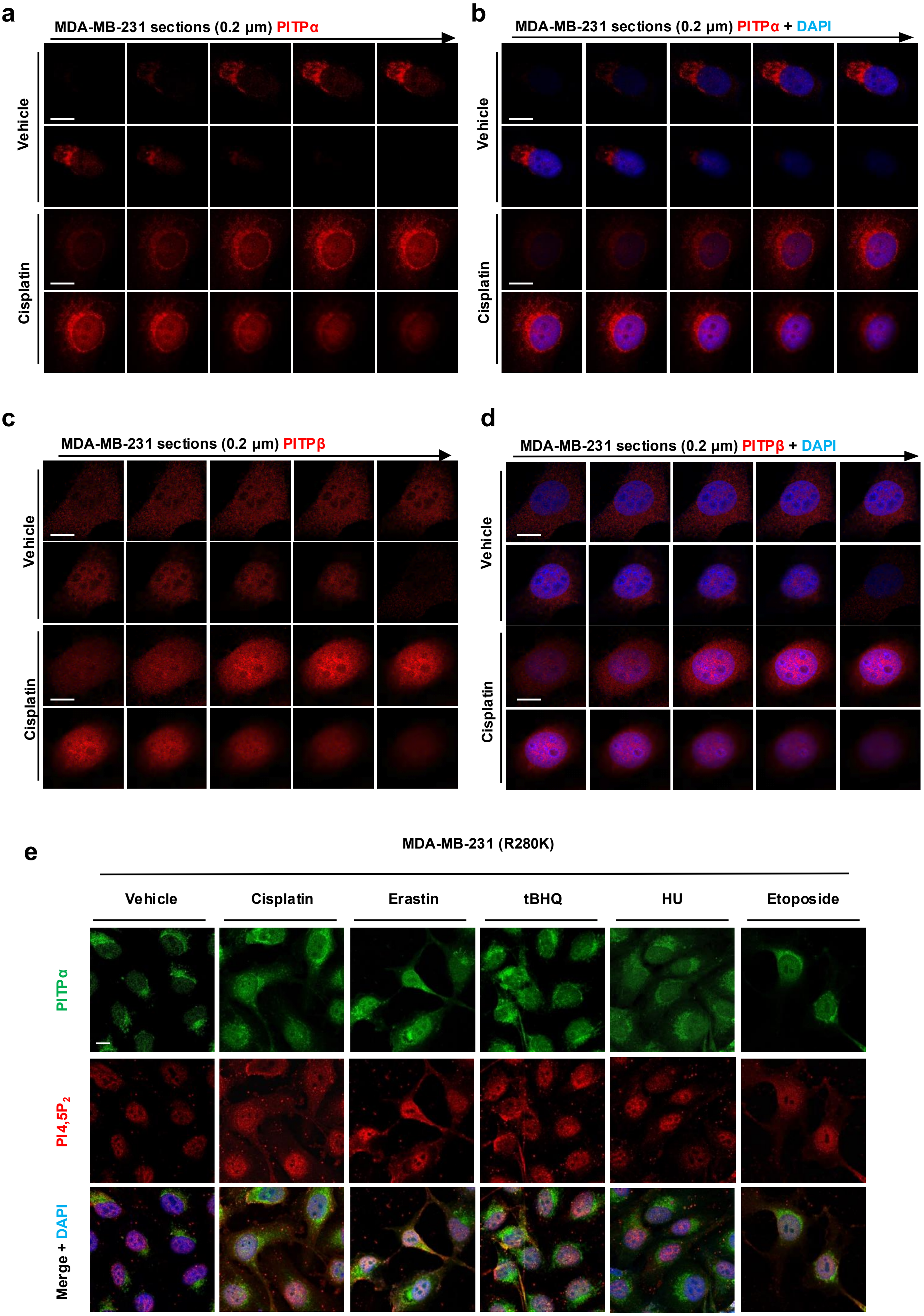

Extended Data Figure 3. 3D sectioning of PITPα/β nuclear accumulation

a-b, 3D sections of IF staining against PITPα in MDA-MB-231 cells treated with vehicle or 30 μM cisplatin for 24 h. The nuclei were counterstained by DAPI. Each frame of the 3D sections was over a 0.2 μm thickness.

c-d, 3D sections of IF staining against PITPβ in MDA-MB-231 cells treated with vehicle or 30 μM cisplatin for 24 h. The nuclei were counterstained by DAPI. Each frame of the 3D sections was over a 0.2 μm thickness.

e, MDA-MB-231 cells treated with 30 μM cisplatin, 20 μM erastin, 100 μM tBHQ, 100 μM etoposide, 100 μM hydroxyurea, or vehicle for 24 h. The cells were processed for IF staining against PITPα and PI4,5P2. The nuclei were counterstained by DAPI. The nuclear levels of PITPα and PI4,5P2 were quantified by ImageJ (**f**). See quantification in Fig. 1f. n=3, 15 cells from each independent experiment.

Scale bar, 5 μm.

Extended Fig. 4

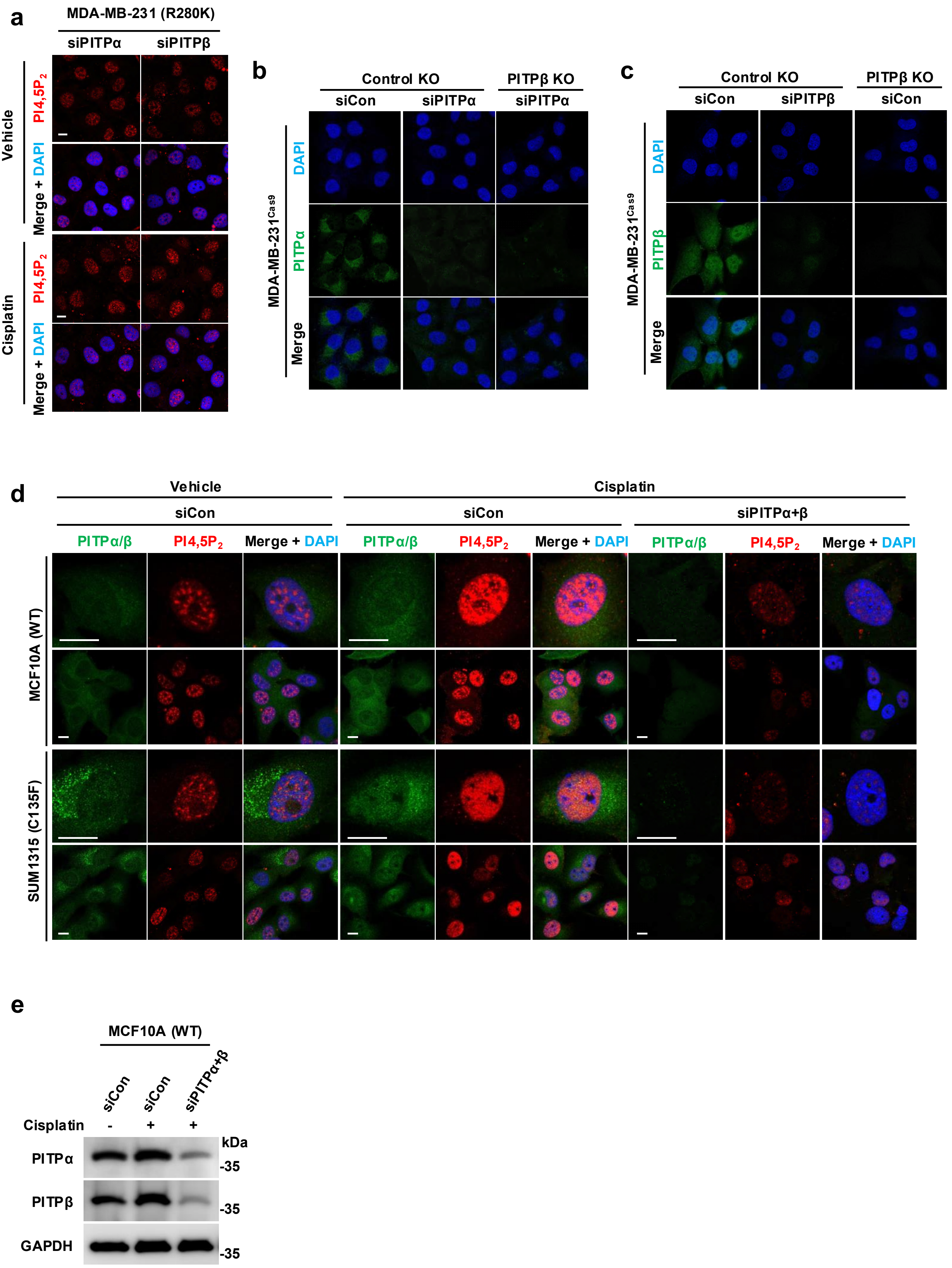

Extended Data Figure 4. PITPα/β are required for nuclear $PI4,5P_2$ pools

a, MDA-MB-231 cells were transfected with siRNAs against PITPα or PITPβ. After 24 h, cells were treated with 30 μM cisplatin or vehicle for 24 h before being processed IF staining against $PI4,5P_2$. The nuclei were counterstained by DAPI. See expanded images in Fig. 2a and quantification in Fig. 2b. n=3 independent experiments.

b-c, MDA-MB-231^{Cas9} cells harboring either a PITPα knockout, PITPβ knockout, or a control knockout were transfected with control siRNA or siRNAs against PITPα or PITPβ. After 48 h cells were processed for IF staining against PITPα or PITPβ. n=3 independent experiments.

d, MCF10A (WT) and SUM1315 (C135F) cells were transfected with control siRNAs or siRNAs against both PITPα and PITPβ. After 24 h, cells were treated with 30 μM cisplatin or vehicle for 24 h before being processed IF staining against $PI4,5P_2$. The nuclei were counterstained by DAPI. See quantification in Fig. 2h. n=3 independent experiments.

Scale bar, 5 μm.

Extended Fig. 5

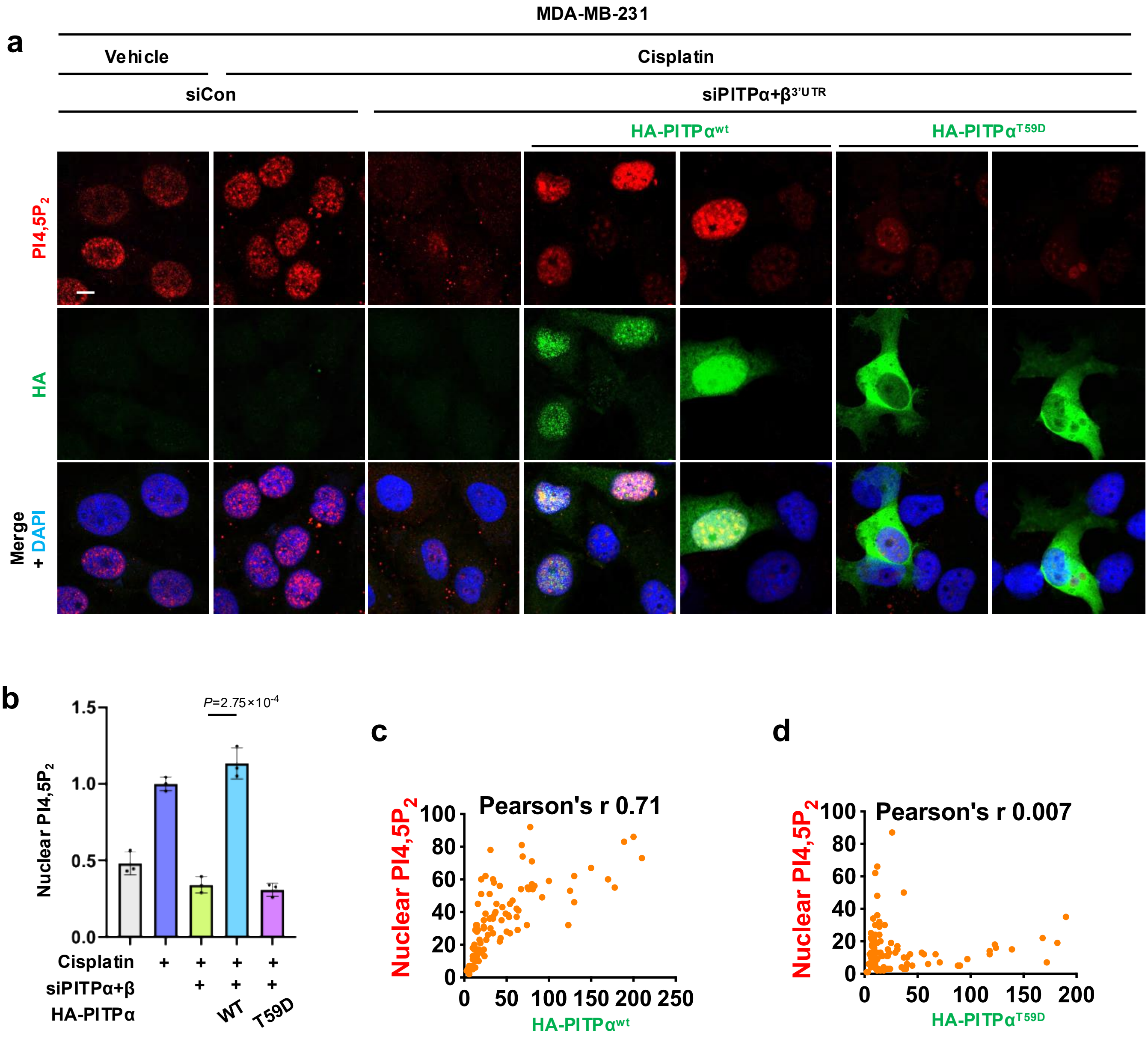

Extended Data Figure 5. PI binding is required for PITP-dependent nuclear $PI4,5P_2$

a-b, MDA-MB-231 cells were transfected with control siRNAs or siRNA against the 3'UTR of both PITPα and PITPβ for 24 h. Then the cells were transfected with either HA-tagged wild-type PITPα or PI-binding defective T59D PITPα. After 24 h, cells were treated with 30 μM cisplatin of vehicle for 24 h before being processed for IF against HA-tag and $PI4,5P_2$ (**a**). The nuclear $PI4,5P_2$ levels in HA-positive cells were quantified by ImageJ (**b**). n=3, average values were calculated from 15 cells from each independent experiment. p value denotes two-sided paired t-test.

c-d, Correlation analysis using LASX between HA-tag (PITPα^{wt} (**c**) or PITPα^{T59D} (**d**)) and $PI4,5P_2$ levels as determined by IF in MDA-MB-231 cells treated with 30 μM cisplatin for 24 h. Pearson's r=0.71 and 0.007 for PITPα^{wt} and PITPα^{T59D}, respectively.

For all graphs, data are presented as the mean ± SD. Scale bar, 5 μm.

Extended Fig. 6

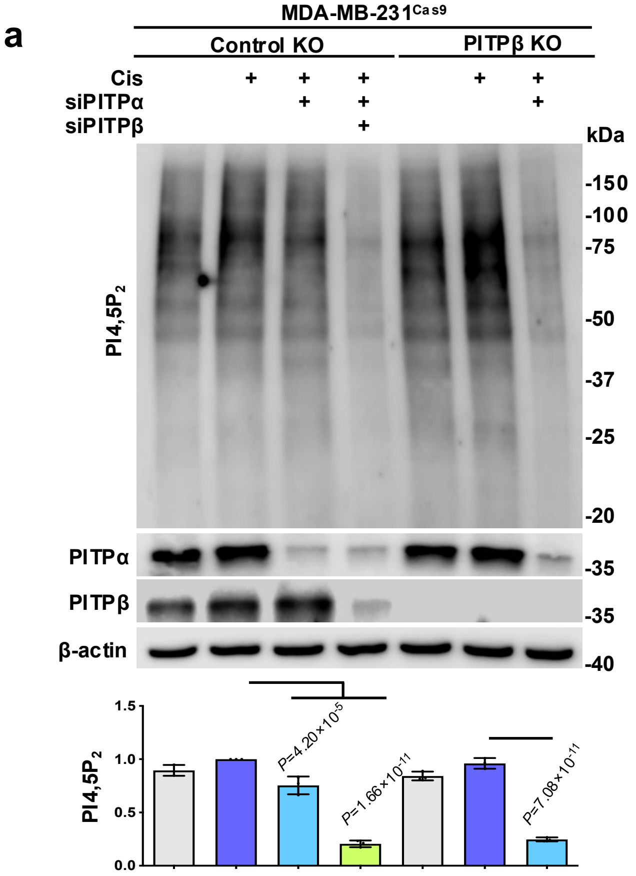

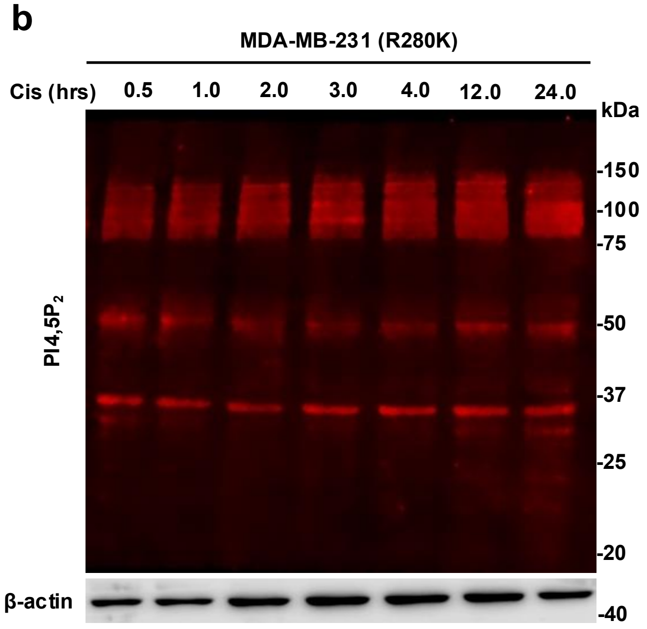

Extended Data Figure 6. PITPα/β regulate stress-responsive, unique, and stable protein-PIP$_n$ complexes

a, MDA-MB-231^{Cas9} cells with PITPβ KO and control non-targeted KO were transfected with control siRNAs or siRNAs against PITPα or both PITPα and PITPβ. After 24 h, cells were treated with 30 μM cisplatin or vehicle for 24 h before being processed for WB against PI4,5P$_2$ and quantified by ImageJ. n=3 independent experiments.

b, MDA-MB-231 cells were treated with 30 μM cisplatin and processed for WB against PI4,5P$_2$ at the indicated treatment timepoints. n=3 independent experiments.

For all graphs, data are presented as the mean ± SD.

Extended Fig. 7

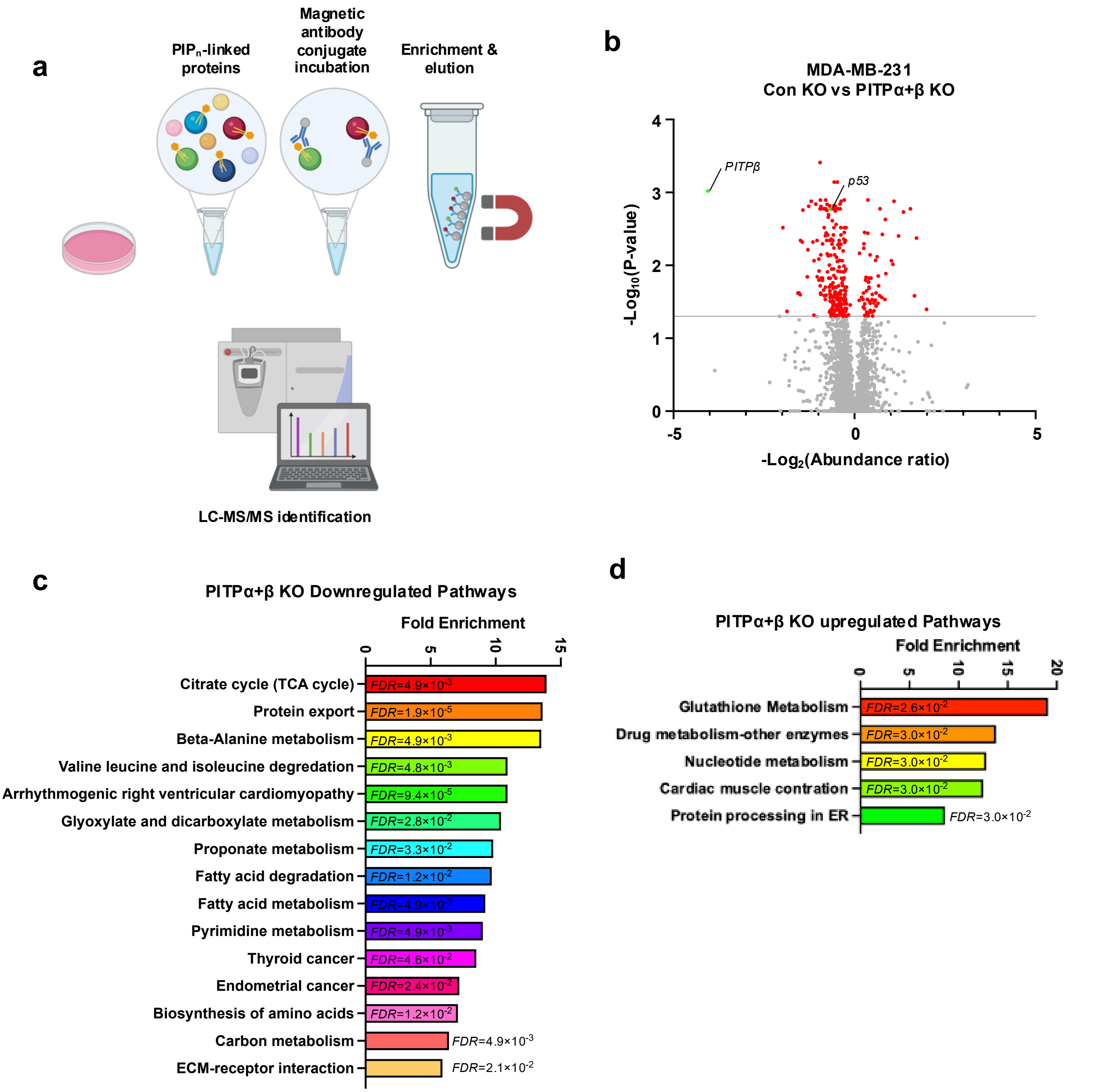

Extended Data Figure 7. Protocol and pathway diagrams from proteomic analysis

a, Graphical diagram depicting IP-MS approach using an anti-PI4,5P_2 antibody and Dynabeads to enrich for protein-PI4,5P_2 complexes.

b-d, MDA-MB-231^{Cas9} cells harboring either a combined PITPα and PITPβ knockout or a control knockout were cultured under normal conditions. Cells were then lysed and proteins were precipitated using chloroform/methanol and resuspended before submitting for mass spectrometry proteomic analysis. All available abundance ratios and corresponding p-values were then graphed (**b**), categorized as either upregulated or downregulated, and submitted for pathway enrichment analysis using ShinyGO 0.85 (**c,d**).
